# High-quality genomic DNA extraction from formalin-fixed and paraffin-embedded samples deparaffinized using mineral oil

**DOI:** 10.1016/j.ab.2009.08.016

**Published:** 2009-12-15

**Authors:** Jianghai Lin, Stephen H. Kennedy, Therese Svarovsky, Jeffrey Rogers, Joseph W. Kemnitz, Anlong Xu, Krina T. Zondervan

**Affiliations:** a Nuffield Department of Obstetrics and Gynaecology, University of Oxford, Oxford, UK; b School of Life Science, Sun Yat-sen University, Guangzhou, China; c Wisconsin National Primate Research Center, University of Wisconsin–Madison, Madison, WI, USA; d Human Genome Sequencing Center, Baylor College of Medicine, Houston, TX, USA; e Wellcome Trust Centre for Human Genetics, University of Oxford, Oxford, UK

## Abstract

Extracting DNA from formalin-fixed and paraffin-embedded (FFPE) tissue remains a challenge, despite numerous attempts to develop a more effective method. Polymerase chain reaction (PCR) success rates with DNA extracted using current methods remain low. We extracted DNA from 140 long-term archived FFPE samples using a simple but effective deparaffinization method, removing the wax with mineral oil, and a commercially available DNA extraction kit. DNA quality was subsequently tested in a genotyping experiment with 14 microsatellite markers. High-quality DNA was obtained with a mean PCR success rate of 97% (range: 88–100%) across markers. The results suggested that DNA extracted using this novel method is likely to be suitable for genetic studies involving DNA fragments <200 bp.

Formalin fixation and paraffin embedding (FFPE)^1^ is a standard method for long-term preservation of most archived pathological specimens. Such samples provide an invaluable resource for subsequent molecular studies of clinical phenotypes, especially genetic studies in which DNA is not available from fresh or frozen tissue(s) because subjects are no longer alive. FFPE tissue is an excellent source of DNA, but its extraction remains a challenge. Formaldehyde, the effective component of formalin, leads to the generation of cross-linking between nucleic acids and proteins [1], and causes nucleic acids to fragment because of fixation process conditions, e.g., the extremely low pH (<1). This makes it very difficult to amplify high molecular weight DNA [2]. Cross-linking not only causes problems in DNA extraction but blocks polymerase chain reaction (PCR) amplification [1]. Despite these problems, FFPE samples are widely used as their DNA content, even in shorter fragments, is often invaluable for studying genetic diseases [1] and is suitable for PCR requiring relatively short DNA fragments [3]. Considerable effort has been made to optimize methods for extracting high-quality DNA from FFPE samples. Indeed, several investigators have successfully extracted genomic DNA stored for 20–25 years by deparaffinizing using xylene/ethanal and extracting DNA using salted-out [4] or phenol purification [2]. Shi et al. [5] suggested that heating FFPE samples at a higher temperature in 0.1 M NaOH solution highly increased the efficiency of DNA extraction. Larger DNA fragments (up to 1182 bp) have been successfully amplified and sequenced from recently stored FFPE tissue (2–4 years) extracted by heating and subsequent use of a modified automatic DNA isolation system [6]. Although several methods for extracting DNA from FFPE specimens are available and widely used, they are generally time-consuming and involve toxic chemicals such as xylene and/or phenol. We describe a novel, simple but effective deparaffinization protocol which, when combined with standard DNA isolation using a commercially available kit, provided high-quality DNA suitable for genotyping experiments.

Archived FFPE tissues (kidney and liver, preserved between 1981 and 2005) were obtained from 140 rhesus monkeys (*Macaca mulatta*) at the Wisconsin National Primate Research Center (WNPRC), Madison, Wisconsin, part of a study into the genetic background of endometriosis [7]. Genomic DNA was extracted from 3 to 4 (5-μm thick) sequential sections for each of the 140 FFPE specimens. Wax was removed from the specimens by adding 300 μl mineral oil to a 1.5-ml microcentrifuge tube containing 3–4 sections of paraffin-embedded tissue, and incubating at 90 °C for 20 min to dissolve the wax. DNA was then extracted with a commercially available kit, DNeasy Blood & Tissue Kit (Qiagen Ltd., West Sussex, UK), using the manufacturer’s protocols, without removing the mineral oil used for deparaffinization, and eluted using 50 μl of elution buffer. The quality of extracted DNA was examined by agarose gel electrophoresis and ethidium bromide staining, and the concentrations were evaluated by OD_260_ (NanoDrop 1000 Spectrophotometers, Thermo Scientific Inc., Waltham, MA).

To test the suitability of the extracted DNA for PCR analysis, DNA from eight FFPE samples were selected across the full range of times since they were first preserved, i.e., in 1982, 1985, 1987, 1990, 1992, 1998, 2001, and 2005, respectively. Beta-actin fragments were amplified by PCR using a set of primers, 1 forward and 5 reverse, producing fragment sizes from 109 to 609 bp (see Supplementary Table), based on the gene sequence downloaded from the Ensembl database: www.ensembl.org (Ref: NP_001028256.1). The PCR system with a final volume of 15 μl contained approximately 25 ng extracted DNA, 0.2 U HotStarTaq DNA polymerase (Qiagen, UK), 0.3 μM each primer, 0.2 μM dNTP mix, and 1.5 mM MgCl_2_. All PCR were performed in a G-Storm GS1 thermal cycler (GRI, UK): 15 min at 95 °C to activate the HotStarTaq DNA polymerase, followed by 45 cycles of denaturation for 45 s, annealing for 45 s, extension for 45 s, and a final extension for 10 min at 72 °C. Amplicons were analyzed on 2% agarose gels stained with ethidium bromide. DNA extracted from FFPE tissues in the beta-actin assays was of sufficient quality for PCR amplification (Fig. 1). All samples, irrespective of the year in which they were fixed and stored, could be amplified effectively up to 191 bp. For the samples fixed and stored in 2001 and 2005, even the 606-bp fragment could be amplified specifically and effectively.

Following the PCR assays with beta-actin, a genotyping experiment was conducted using DNA from all 140 FFPE samples, involving 14 microsatellite markers across two regions orthologous to human chromosomes 7 and 10 (previously linked to endometriosis in women [9,10]). Primers were designed using Primer 3 [8] to ensure that all amplicons were approximately 100 bp in length so that most samples could be amplified successfully. The 5′ terminals of each forward marker were labeled with a fluorescent dye (6FAM, VIC, NED, or PET) for multiplex genotyping. DNA samples from all FFPE specimens were included in the same PCR system, with reaction conditions as described above, and the MgCl_2_ concentration was adjusted for different markers (from 1.5 to 2.5 M). The marker PCR amplicons were analyzed using an ABI 3700 DNA analyzer (Applied Biosystems, Foster City, CA). Sample genotypes were called by Genemapper v3.7 (Applied Biosystems). As shown in Table 1, for each of the markers, PCR and genotyping were successful for 88–100% of the specimens, with an average success rate of 97% across the 14 markers. To investigate the effect of year of archiving, PCR success rate was also calculated across all markers for each individual sample. The average yearly PCR success rate was significantly correlated with year of archiving (Spearman *ρ* = 0.25, *P* = 0.003; Supplementary Figure). However, this correlation was driven by the 100% PCR success rates in the samples archived for less than 10 years (*n* = 20); when limiting the analysis to years 1980–1995, there was no significant correlation.

To the best of our knowledge, this is the first report of the use of mineral oil as a deparaffinizing reagent. Both mineral oil and paraffin have been used in PCR for many years, and it was already known that neither affect the efficiency of PCR; moreover, paraffin can be dissolved in mineral oil completely. For these reasons, we hypothesized that mineral oil could be used to remove wax effectively from FFPE samples without affecting DNA quality for downstream PCR and genotyping experiments. One concern regarding this simple method was that the fixation reagent, formalin, might not be removed during the deparaffinizing process and, if not, it might affect the efficiency of the PCR. However, in our genotyping experiments no adverse effects were observed.

Vast numbers of FFPE tissues are being kept in pathology department archives worldwide as it is a standard preservation method in clinical practice. These samples represent an invaluable DNA bank for genetic studies. An effective method for DNA extraction that involves minimum manipulation is required for treating such specimens. Compared with the most commonly adopted xylene/ethanol method, the approach described here is very simple and effective. The observed minimum success rate of 88% for 140 samples tested using 14 microsatellite markers was considerably higher than some reported results: 69% was achieved in a xylene/phenol method with sample archived for 20–25 years [2] and 75% using a commercial kit [11]. Our results showed that our method can also be used on samples stored over a long period—over 25 years in some cases—as long as large fragments of DNA molecules are not required. DNA extracted from FFPE specimens usually only allows PCR analysis on relatively short target sequences, rarely exceeding 300 bp [2], because of DNA fragmentation during the processes of fixation and embedding. The results of DNA extraction in the current study agreed with this observation: the majority of the fragments were approximately 200 bp in length (data not shown). The results of the beta-actin assays and the correlation analysis between archiving year and PCR success rate indicated that the storage time might significantly affect the DNA integrity and PCR amplification. Therefore, DNA samples extracted from FFPE tissues stored for long periods of time may not be suitable for analysis if longer DNA molecules are needed, as in resequencing studies. However, shorter fragment DNA isolated from FFPE tissue samples may be suitable for most genetic analyses that require only short DNA molecules (i.e., single nucleotide polymorphism, microsatellite, and methylation status analyses).

It is worth noting that the integrity of DNA is critically affected by the fixation method, i.e., the fixative and process of fixation, used when preserving tissues [2]. Lydidis and colleagues [12] recently published a modified zinc-based fixation method which greatly increase the quality and quantity of DNA recovered from preserved tissues stored for up to 14 months. This indicates that improvement in the fixation process of tissue preservation will also help a lot in recovering genetic material of archived samples in the future.

In summary, the novel deparaffinizing method with mineral oil introduced here is simple and effective. Our results suggest that in combination with commercially available DNA extraction kits, this method could substantially improve the efficiency of DNA recovery experiments from FFPE specimens, even when archived for as long as 27 years.

## Figures and Tables

**Fig. 1:**
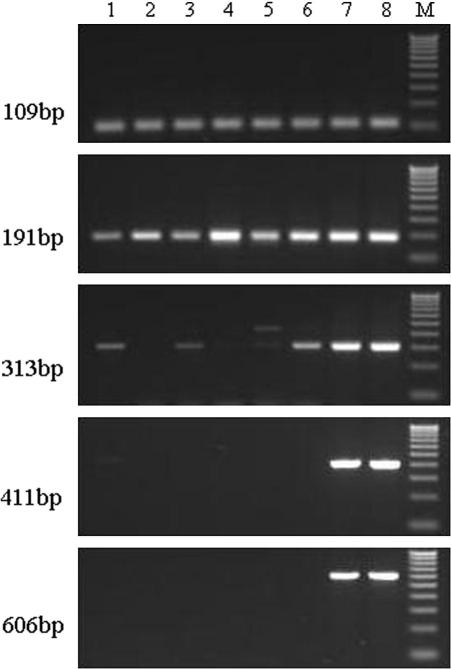
PCR analysis of β-actin with five different primer sets. The size of PCR products is indicated on the left of the figure. Numbers 1–8 refer to samples stored in 1982, 1985, 1987, 1990, 1992, 1998, 2001 and 2005 respectively; M is 100 bp ladder.

**Table 1:** PCR results of 140 DNA samples extracted from FFPE specimens, using 14 microsatellite markers.

Markers	Product size (bp)	Number of successfully amplified/genotyped samples (%)
D7S497	95–107	140 (100%)
D7S2427	79–116	140 (100%)
D7S2428	72–110	130 (92.9%)
MML3S40	100–110	140 (100%)
MML3S42	99–125	133 (95.0%)
MML3S43	98–112	139 (99.3%)
MML3S44	89–100	137 (97.9%)
D10S216	99–126	123 (87.9%)
D10S1679	93–125	135 (96.4%)
D10S1723	77–121	136 (97.1%)
D10S575	105–140	134 (95.7%)
MML9S29	104–128	132 (94.3%)
MML9S30	74–99	140 (100%)
MML9S32	97–115	136 (97.1%)

## Appendix A Supplementary data

Supplementary Table PCR primers for beta-actin.

## Footnotes

1 *Abbreviations used*: FFPE, formalin fixed and paraffin embedded; PCR, polymerase chain reaction.
