# Supplementary material for: High-quality genomic DNA extraction from formalin-fixed and paraffin-embedded samples deparaffinized using mineral oil
Source: Anal Biochem. 2009 Dec 15;395(2):265–7. doi: 10.1016/j.ab.2009.08.016 (PMC2764035; doi:10.1016/j.ab.2009.08.016)
Supplement: Supplementary Table — PCR primers for beta-actin. [file mmc1.doc]

Supplementary Table: PCR primers for beta-actin

| Primer | Primer Sequence | Product size (bp) | °C^1^ |
| --- | --- | --- | --- |
| Forward | CACACTGTGCCCATCTATGAGG | -- | 50 |
| Reverse 1 | CGCGCTCAGTGAGGATCTTC | 109 | 50 |
| Reverse 2 | TCGAAGTCTAGGGCGACATAGC | 191 | 50 |
| Reverse 3 | AAGGTTGGAAGAGAGCCTCAGG | 313 | 50 |
| Reverse 4 | GAAGAAATGAGGGCGGACTTAG | 411 | 50 |
| Reverse 5 | ACACCCACCTTGATCTTCATCG | 606 | 55 |

1: annealing temperature

Supplementary Figure: Average PCR success rates (with standard errors) across 140 samples according to archiving year.
